# Supplementary material for: Hot spot prediction in protein-protein interactions by an ensemble system
Source: BMC Syst Biol. 2018 Dec 31;12(Suppl 9):132. doi: 10.1186/s12918-018-0665-8 (PMC6311905; doi:10.1186/s12918-018-0665-8)
Supplement: Supplementary file 2 — All relevant MATLAB code. A simple Matlab implement of our predictor. (ZIP 19,742 kb) [file 12918_2018_665_MOESM2_ESM.zip › Additional file 2/Read ASA/ASA website/SKEMPI/2QJ9C.htm]

 NetSurfP - Protein Surface Accessibility and Secondary Structure Predictions

  

|  |  |  |
| --- | --- | --- |
|  |  | NetSurfP - Protein Surface Accessibility and Secondary Structure Predictions  Technical University of Denmark |

  


---

```
# For publication of results, please cite:
# A generic method for assignment of reliability scores applied to solvent accessibility predictions.
# Bent Petersen, Thomas Nordahl Petersen, Pernille Andersen, Morten Nielsen and Claus Lundegaard
# BMC Structural Biology 2009, 9:51 doi:10.1186/1472-6807-9-51
#
# Column 1: Class assignment - B for buried or E for Exposed - Threshold: 25% exposure, but not based on RSA
# Column 2: Amino acid
# Column 3: Sequence name
# Column 4: Amino acid number
# Column 5: Relative Surface Accessibility - RSA
# Column 6: Absolute Surface Accessibility
# Column 7: Z-fit score for RSA prediction
# Column 8: Probability for Alpha-Helix
# Column 9: Probability for Beta-strand
# Column 10: Probability for Coil
E T  2QJ9C                  1    0.855 118.644   0.480   0.003   0.003   0.994
B L  2QJ9C                  2    0.268  49.126  -0.293   0.004   0.085   0.910
E P  2QJ9C                  3    0.350  49.679  -0.552   0.019   0.141   0.840
B F  2QJ9C                  4    0.241  48.449   0.098   0.004   0.420   0.576
B L  2QJ9C                  5    0.059  10.785  -0.837   0.002   0.816   0.182
E K  2QJ9C                  6    0.292  60.003   0.903   0.001   0.900   0.099
B C  2QJ9C                  7    0.021   2.920   0.451   0.001   0.900   0.099
B Y  2QJ9C                  8    0.252  53.917   1.107   0.001   0.900   0.099
B C  2QJ9C                  9    0.020   2.752   0.848   0.004   0.616   0.381
E S  2QJ9C                 10    0.309  36.273   0.720   0.004   0.138   0.858
E G  2QJ9C                 11    0.648  50.966  -2.606   0.018   0.088   0.893
E H  2QJ9C                 12    0.495  90.131  -0.004   0.019   0.141   0.840
B C  2QJ9C                 13    0.043   6.093  -0.326   0.018   0.088   0.893
E P  2QJ9C                 14    0.573  81.280  -0.378   0.018   0.047   0.935
E D  2QJ9C                 15    0.636  91.633  -2.045   0.018   0.019   0.964
E D  2QJ9C                 16    0.515  74.284  -1.725   0.018   0.019   0.964
E A  2QJ9C                 17    0.426  46.989  -1.457   0.018   0.019   0.964
E I  2QJ9C                 18    0.530  98.087  -0.926   0.018   0.047   0.935
E N  2QJ9C                 19    0.421  61.590  -1.309   0.018   0.047   0.935
B N  2QJ9C                 20    0.245  35.868  -1.242   0.018   0.088   0.893
E T  2QJ9C                 21    0.282  39.113   0.850   0.022   0.359   0.619
B C  2QJ9C                 22    0.039   5.532  -0.665   0.022   0.552   0.426
E I  2QJ9C                 23    0.395  73.131   0.929   0.069   0.386   0.545
E T  2QJ9C                 24    0.486  67.353  -0.906   0.056   0.142   0.802
E N  2QJ9C                 25    0.605  88.499  -0.900   0.053   0.043   0.903
B G  2QJ9C                 26    0.260  20.486  -0.879   0.053   0.043   0.903
E H  2QJ9C                 27    0.289  52.624   0.778   0.074   0.484   0.442
B C  2QJ9C                 28    0.033   4.661   0.160   0.021   0.756   0.223
B F  2QJ9C                 29    0.064  12.885   0.900   0.018   0.846   0.136
B A  2QJ9C                 30    0.140  15.472   0.801   0.018   0.846   0.136
B I  2QJ9C                 31    0.091  16.853   0.441   0.018   0.846   0.136
B I  2QJ9C                 32    0.084  15.614   0.575   0.011   0.918   0.071
B E  2QJ9C                 33    0.227  39.622  -0.112   0.011   0.918   0.071
E E  2QJ9C                 34    0.322  56.271   0.368   0.011   0.918   0.071
B D  2QJ9C                 35    0.225  32.480  -0.288   0.023   0.655   0.322
E D  2QJ9C                 36    0.623  89.731  -0.394   0.022   0.359   0.619
E Q  2QJ9C                 37    0.679 121.269  -1.057   0.018   0.088   0.893
E G  2QJ9C                 38    0.365  28.757  -0.882   0.018   0.088   0.893
E E  2QJ9C                 39    0.415  72.431  -0.235   0.023   0.655   0.322
E T  2QJ9C                 40    0.358  49.668   0.403   0.011   0.918   0.071
E T  2QJ9C                 41    0.278  38.489   0.435   0.006   0.962   0.032
E L  2QJ9C                 42    0.256  46.892   1.140   0.011   0.918   0.071
B A  2QJ9C                 43    0.311  34.261  -0.472   0.018   0.846   0.136
B S  2QJ9C                 44    0.197  23.124   0.323   0.004   0.616   0.381
B G  2QJ9C                 45    0.221  17.408  -1.545   0.005   0.336   0.660
B C  2QJ9C                 46    0.055   7.764  -1.089   0.005   0.262   0.733
B M  2QJ9C                 47    0.123  24.512  -0.252   0.019   0.141   0.840
E K  2QJ9C                 48    0.363  74.628   1.060   0.018   0.047   0.935
E Y  2QJ9C                 49    0.366  78.193   0.078   0.018   0.019   0.964
E E  2QJ9C                 50    0.647 113.118  -1.652   0.018   0.019   0.964
E G  2QJ9C                 51    0.639  50.329  -2.287   0.018   0.019   0.964
E S  2QJ9C                 52    0.419  49.083  -0.951   0.018   0.047   0.935
E D  2QJ9C                 53    0.383  55.205   0.160   0.021   0.279   0.699
B F  2QJ9C                 54    0.070  14.149  -0.623   0.021   0.451   0.528
B Q  2QJ9C                 55    0.242  43.168   0.049   0.022   0.552   0.426
B C  2QJ9C                 56    0.026   3.622  -1.022   0.022   0.552   0.426
E R  2QJ9C                 57    0.351  80.264   0.036   0.005   0.336   0.660
E D  2QJ9C                 58    0.411  59.240  -1.247   0.004   0.085   0.910
E T  2QJ9C                 59    0.538  74.579  -0.381   0.018   0.088   0.893
E P  2QJ9C                 60    0.445  63.089  -1.692   0.018   0.047   0.935
E I  2QJ9C                 61    0.471  87.043  -0.268   0.018   0.019   0.964
E P  2QJ9C                 62    0.399  56.646  -1.603   0.018   0.019   0.964
E H  2QJ9C                 63    0.491  89.331  -1.357   0.018   0.019   0.964
E Q  2QJ9C                 64    0.481  85.960  -0.718   0.018   0.019   0.964
E R  2QJ9C                 65    0.438 100.394  -1.065   0.018   0.047   0.935
E R  2QJ9C                 66    0.318  72.799   0.110   0.021   0.279   0.699
B S  2QJ9C                 67    0.269  31.515   0.500   0.021   0.756   0.223
B I  2QJ9C                 68    0.079  14.541   1.023   0.018   0.846   0.136
B E  2QJ9C                 69    0.259  45.230   0.707   0.018   0.846   0.136
B C  2QJ9C                 70    0.026   3.693   0.446   0.023   0.655   0.322
B C  2QJ9C                 71    0.062   8.775   0.816   0.021   0.451   0.528
E R  2QJ9C                 72    0.487 111.523   0.110   0.018   0.088   0.893
E T  2QJ9C                 73    0.536  74.329   0.076   0.018   0.019   0.964
E N  2QJ9C                 74    0.591  86.464  -1.032   0.058   0.017   0.925
B L  2QJ9C                 75    0.212  38.854   0.657   0.115   0.016   0.868
B C  2QJ9C                 76    0.046   6.416  -0.358   0.115   0.016   0.868
B N  2QJ9C                 77    0.260  38.020  -0.454   0.115   0.016   0.868
E Q  2QJ9C                 78    0.551  98.480  -0.348   0.053   0.043   0.903
E Y  2QJ9C                 79    0.416  89.006  -0.366   0.018   0.047   0.935
B L  2QJ9C                 80    0.083  15.234   0.284   0.018   0.047   0.935
E Q  2QJ9C                 81    0.388  69.279   0.717   0.005   0.045   0.951
B P  2QJ9C                 82    0.243  34.538  -0.909   0.005   0.045   0.951
E T  2QJ9C                 83    0.597  82.790  -0.908   0.005   0.045   0.951
B L  2QJ9C                 84    0.213  39.074   0.283   0.005   0.015   0.979
E P  2QJ9C                 85    0.460  65.217   0.256   0.003   0.003   0.994
E P  2QJ9C                 86    0.789 111.973  -1.742   0.003   0.003   0.994

Explain the output.  Go back.
```

---
